# Supplementary material for: EnzML: multi-label prediction of enzyme classes using InterPro signatures
Source: BMC Bioinformatics. 2012 Apr 25;13:61. doi: 10.1186/1471-2105-13-61 (PMC3483700; doi:10.1186/1471-2105-13-61)
Supplement: Addtional file 5 — The Java code to format the data files, evaluate and predict. The file enzml_java_code.tar.gz contains the Java code used to format database data to ARFF and XML formats, to execute cross and train-test (jackknife) evaluations and to record evaluation results to database. More information is included in the readme.txt file and the Javadoc files. The code can be used with a MySQL database. To use a different database software, other JDBC drivers might be required. [file 1471-2105-13-61-S5.gz › java_code/utils/doc/uk/ac/ed/inf/utils/package-summary.html]

uk.ac.ed.inf.utils


---


|  |  |  |
| --- | --- | --- |
| **Overview** \| **Package** \| Class \| **Use** \| **Tree** \| **Deprecated** \| **Index** \| **Help** | |  |
| **PREV PACKAGE**   **NEXT PACKAGE** | **FRAMES**    **NO FRAMES**     **All Classes** |  |


---

## Package uk.ac.ed.inf.utils

| **Interface Summary** | |
| --- | --- |
| **Initialisable** | A class having a boolean variable telling whether the initialisation has completed correctly |

| **Class Summary** | |
| --- | --- |
| **ArrayUtils** | Utilities to manipulate arrays. |
| **CollectionUtils** | Class |
| **EntrezUtils** | Utilities for Entrez services. |
| **FileUtils** | Utils to read, write files etc. |
| **Initialised** |  |
| **ListUtils** | Utilities to manipulate lists |
| **LoggerCreator** |  |
| **LogUtils** | Utilities to log error messages |
| **NumberUtils** | Class |
| **PathUtils** | Paths to utilities folders and files |
| **PropertiesUtils** | Utilities to manipulate Java properties and properties text files |
| **ReflectionUtils** | Class |
| **RegExpUtils** |  |
| **SetUtils** | Class |
| **StringUtils** | Class |
| **TimeUtils** | Utilities to manipulate time objects, create timestamps etc. |
| **Utils** | Various utilities to manipulate vectors of numbers, get a timestamp etc. |
| **VectorUtils** |  |

---


|  |  |  |
| --- | --- | --- |
| **Overview** \| **Package** \| Class \| **Use** \| **Tree** \| **Deprecated** \| **Index** \| **Help** | |  |
| **PREV PACKAGE**   **NEXT PACKAGE** | **FRAMES**    **NO FRAMES**     **All Classes** |  |


---
